# Combined sonographic optic nerve sheath diameter and cerebral oximeter for predicting neurological outcome after cardiac arrest

**DOI:** 10.17305/bb.2024.11442

**Published:** 2024-11-20

**Authors:** Mehmet Akif Yazar, Betul Kozanhan, Yasin Tire, Nevin Sekmenli, Guzide Yazar, Murat Sevim

**Affiliations:** 1 Outcome Research Consortium, Cleveland Clinic, Cleveland, OH, USA; 2 University of Health Sciences, Konya City Hospital, Department of Anesthesiology and Reanimation, Konya, Türkiye; 3 University of Health Sciences, Konya City Hospital, Department of Radiology, Konya, Türkiye

**Keywords:** Optic nerve sheath diameter, ONSD, near-infrared spectroscopy, NIRS, neurological outcome, post-cardiac arrest, CA

## Abstract

Cardiac arrest (CA) remains a critical global health issue with high rates of mortality and morbidity. Accurate prediction of neurological outcomes in post-CA patients is essential for optimizing management strategies. Optic nerve sheath diameter (ONSD) and near-infrared spectroscopy (NIRS) are emerging as promising tools for evaluating brain oxygenation and intracranial pressure (ICP). However, the potential benefits of combining these methods for improved prognostic accuracy have not been thoroughly explored. This study investigates whether the combined use of ultrasonographic ONSD and NIRS measurements enhances the prediction of neurological outcomes after CA. In this prospective study, ONSD measurements were obtained three times at 24-h intervals, while regional hemoglobin oxygen saturation (rSO2) using NIRS was recorded twice. Neurological outcomes were assessed using the Full Outline of Unresponsiveness (FOUR) and Cerebral Performance Categories (CPC) scores for both early and late evaluations. Results indicated that 47.5% of patients had poor outcomes and 52.5% had good outcomes based on the FOUR score, while 65% had poor outcomes and 35% had good outcomes according to the CPC score. The combination of ONSD and NIRS measurements showed superior prognostic performance compared to either method alone. While standalone NIRS measurements taken after 24 h exhibited limited predictive value, combining ONSD and NIRS provided a more reliable approach for neurological assessment in the short term following CA. This integrated method may improve prognostic accuracy and support better clinical decision making.

## Introduction

Cardiac arrest (CA) is a global health issue with high mortality and morbidity rates. Each year, millions of individuals experience CA, but only a small percentage achieve meaningful neurological recovery due to severe anoxic brain injury. Following CA, cerebral injury from prolonged anoxic perfusion often results in significant neurological deficits. These deficits are further compounded by delays in restoring effective cerebral perfusion during both the arrest and post-resuscitation phases. The return of spontaneous circulation (ROSC) after CA frequently leads to acute brain edema and elevated intracranial pressure (ICP), caused by ischemia-reperfusion injury and delayed hyperemia [1]. This cascade of events severely impacts the quality of life for many CA survivors, who often face cognitive and motor impairments to varying degrees. Early intervention and accurate prognosis are critical for guiding care and optimizing rehabilitation outcomes in this vulnerable population.

Early and accurate prediction of poor neurological outcomes in CA survivors is crucial, as it allows healthcare providers to avoid futile treatments for patients with poor prognoses. Timely prognostication enables a shift in focus toward implementing palliative measures that optimize comfort and quality of life. Conversely, patients with indicators of a favorable prognosis can receive more aggressive and targeted interventions aimed at recovery. Identifying comprehensive and necessary treatment protocols that may benefit these patients is therefore of paramount importance.

Various methods have been proposed for assessing neurological outcomes, ranging from advanced imaging techniques to bedside clinical assessments. Recently, some studies have explored the early prediction of neurological outcomes by evaluating the optic nerve sheath diameter (ONSD) in CA patients [2]. Due to the optic nerve’s anatomical continuity with the brain and its sensitivity to ICP fluctuations, ONSD has emerged as a potentially valuable marker for assessing cerebral edema and elevated ICP. Previous studies have suggested that ONSD, along with the gray–white matter ratio (GWR) on computed tomography (CT), may serve as important indicators for evaluating acute brain edema and predicting neurological outcomes [3, 4]. This association highlights the relevance of non-invasive measures for assessing brain injury, as they can provide real-time insights into a patient’s intracranial status without requiring transport or exposing the patient to radiation.

In addition to CT, ONSD can be easily measured using bedside ultrasonography. Ultrasonography offers several advantages over CT, including portability, the absence of radiation exposure, and the ability to perform serial measurements. These features make it a valuable tool in critical care settings, where immediate and repeated assessments are often necessary. Numerous studies have demonstrated a strong relationship between increased ICP and sonographic ONSD measurements [5, 6]. However, it is important to note that the utility of ONSD in predicting long-term neurological outcomes remains under investigation. Some studies suggest that ONSD measurements may not correlate with outcomes at six months post-ROSC [7]. This discrepancy underscores the complexity of brain injury following CA and highlights the potential need for multimodal approaches to enhance prognostic accuracy.

Near-Infrared Spectroscopy (NIRS) measures total oxygen saturation in a given tissue volume by estimating the oxygen saturation of the hemoglobin fraction within the terminal vasculature. This technique allows for the noninvasive measurement of regional oxygen saturation (rSO2) in brain tissue [8, 9]. Unlike systemic oxygenation metrics, which may not accurately reflect cerebral oxygenation, NIRS provides region-specific insights into brain tissue oxygenation. This is particularly crucial in the post-CA period, where cerebral hypoxia and ischemia are common.

Post-hypoxic brain injury following CA often involves changes in oxygen consumption, cerebral blood flow, or cerebral blood volume, all of which may influence rSO2 in brain tissue. Continuous bedside monitoring of frontal brain rSO2 using NIRS could offer valuable information for early neurological outcome prediction. NIRS has the distinct advantage of enabling real-time, continuous monitoring, allowing clinicians to track oxygenation trends and adjust interventions as needed.

However, while NIRS shows promise, some studies indicate that its prognostic reliability is limited [10]. The variability in NIRS findings may stem from individual differences in cerebral autoregulation, the timing of measurements, or the impact of confounding factors such as hemodynamic instability or sedation.

By combining bedside noninvasive measurements, such as ONSD and NIRS, the early prediction of neurological outcomes CA patients with ROSC could significantly influence ICU treatment strategies, potentially leading to better outcomes. Integrating these methods may enhance prognostic accuracy compared to using either technique alone, offering a more comprehensive assessment of brain injury in CA survivors. This innovative approach holds promise for improving clinical decision making by guiding the intensity and duration of therapeutic interventions in the ICU.

In this study, the prognostic value of combining ultrasonographic ONSD and NIRS measurements was investigated to predict neurological outcomes CA. This integrated approach aims to provide clinicians with a more robust prognostic framework, potentially minimizing unnecessary treatments for patients with poor outcomes while optimizing care for those with better recovery prospects. By highlighting the synergistic value of these two non-invasive monitoring techniques, the study seeks to offer novel insights into neurological prognostication for post-CA patients. While ONSD and NIRS have individually shown promise, their combined use remains underexplored, particularly in real-world ICU settings. This research addresses a critical gap in the literature by testing the hypothesis that combining ONSD and NIRS will yield superior prognostic accuracy compared to using either modality alone.

## Materials and methods

### Study design and subjects

**Figure 1.**
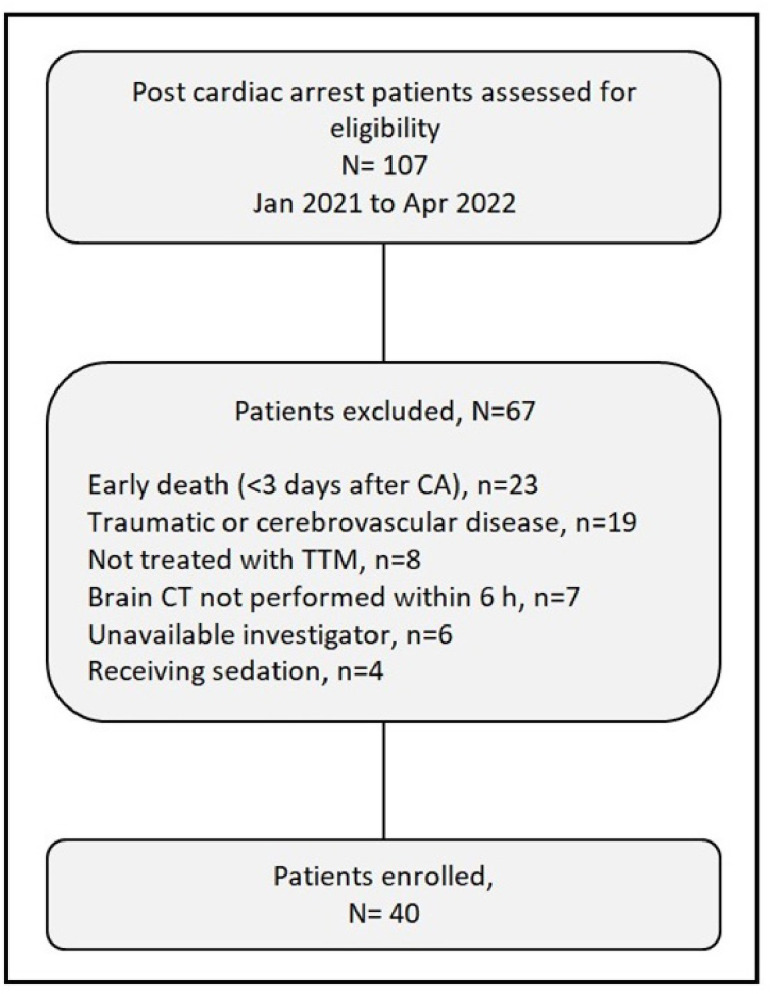
**Flowchart of study selection.** TTM: Targeted temperature management; CT: Computed tomography; CA: Cardiac arrest.

Between January 2021 and April 2022, 107 patients were assessed, and 40 were ultimately enrolled in this prospective cohort observational study. A detailed flowchart of the study is provided in Figure 1. Due to the nature of the cohort study and its time constraints, a formal sample size calculation was not performed.

The study included patients who met the following criteria: ICU admission following CA, whose post-arrest GCS was ≤6, who were ≥18 years, who had a brain tomography performed within the first 6 h after CA, treatment with targeted temperature management (TTM), and no sedation.

**Figure 2.**
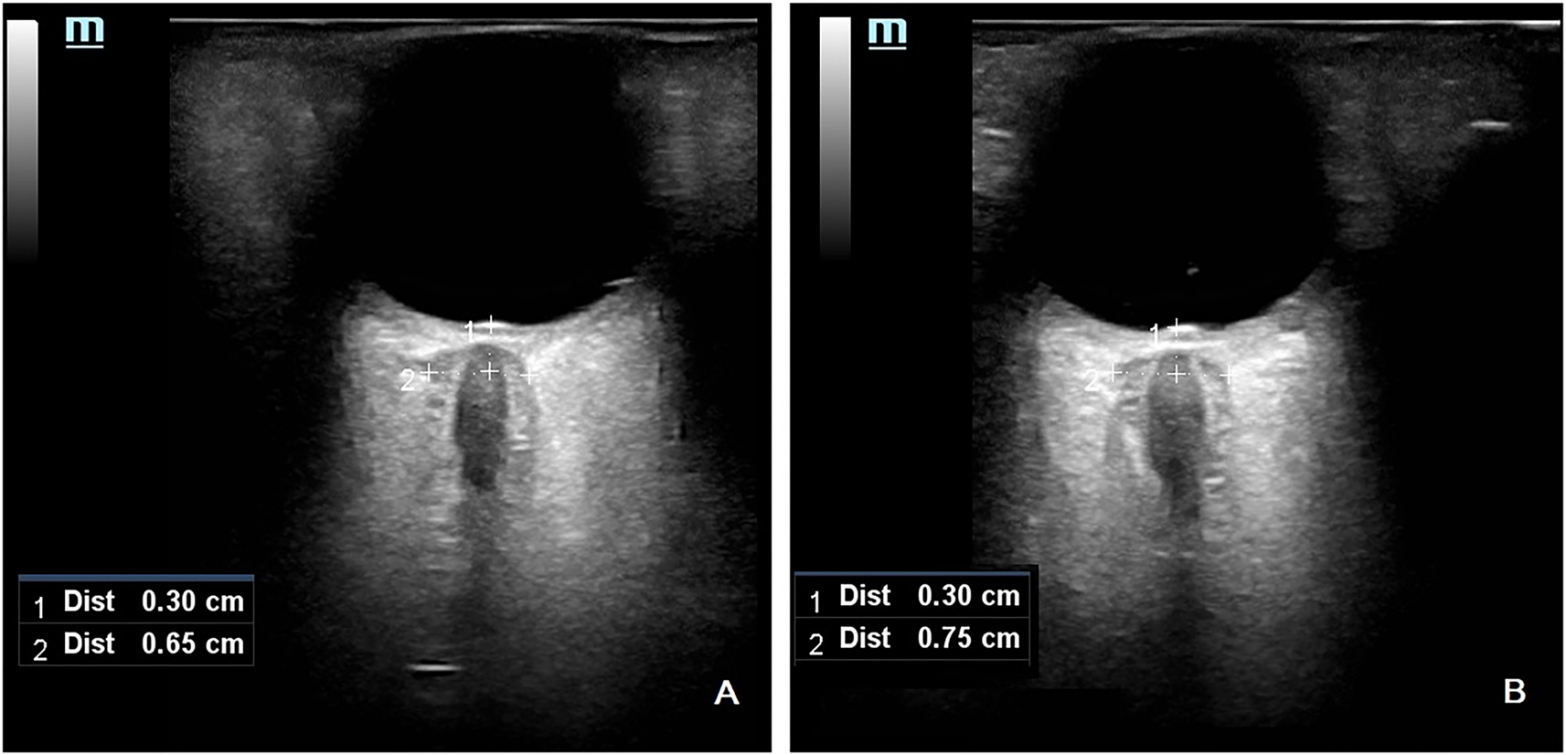
**Ultrasonographic ONSD measurement.** (A) Ultrasonographic ONSD measurement of patient number 11; (B) Ultrasonographic ONSD measurement of patient number 32. ONSD: Optic nerve sheath diameter.

Patients were excluded if any of the following applied: measurements could not be completed within the first 24 h after CA (e.g., due to early death, severe hemodynamic instability, or the unavailability of devices or investigators on weekends), CA caused by intracranial trauma or a neurological event, a history of cerebrovascular accident, orbital or eyeball injuries, facial trauma, ocular pathologies such as exophthalmos, glaucoma, or cataracts, ongoing sedation or a need for sedation, or recurrent CA within three days of ICU admission.

### Post-resuscitation and TTM protocol

All patients who experienced in-hospital or out-t CA were treated according to the advanced life support protocols outlined in the European Resuscitation Council (ERC) 2015 guidelines. Upon arrival in the ICU, patients received TTM and intensive care in alignment with our hospital’s ICU protocol. Hypothermia was induced using a surface-cooling device and blankets (Arctic Sun^®^ Energy Transfer Pads™ , Medivance Corp., Louisville, USA). The target temperature of 34 ^∘^C was maintained for 24 h, followed by controlled rewarming to 36.5 ^∘^C at a rate of 0.15 ^∘^C per hour, with temperature monitoring conducted via an esophageal probe.

### Measurement of ultrasonographic ONSD

The measurement of ONSD was conducted as described in the supplemental digital content, following protocols from previous studies [11]. For each patient, ONSD was measured upon arrival at the ICU and subsequently at two 24-h intervals (designated as ONSD1, ONSD2, and ONSD3, respectively). Two radiologists, experienced in transorbital ultrasonography and blinded to the patients’ clinical courses, performed the measurements. To measure ONSD, a thick layer of ultrasound gel was applied over the closed upper eyelid, and a 12-MHz ultrasonographic probe (Toshiba XarioR) was positioned over the temporal region of the eyelid. Care was taken to apply minimal pressure to the eye during the examination. The imaging depth was adjusted to 4 cm, and the 2D mode was employed. For safety reasons concerning potential biomechanical side effects, the mechanical index was reduced to 2. ONSD was measured 3 mm behind the papilla of each eye, between the external hypoechogenic margins, using an electronic caliper aligned perpendicularly to the optic nerve axis (Figure 2).

### Brain CT scan

**Figure 3.**
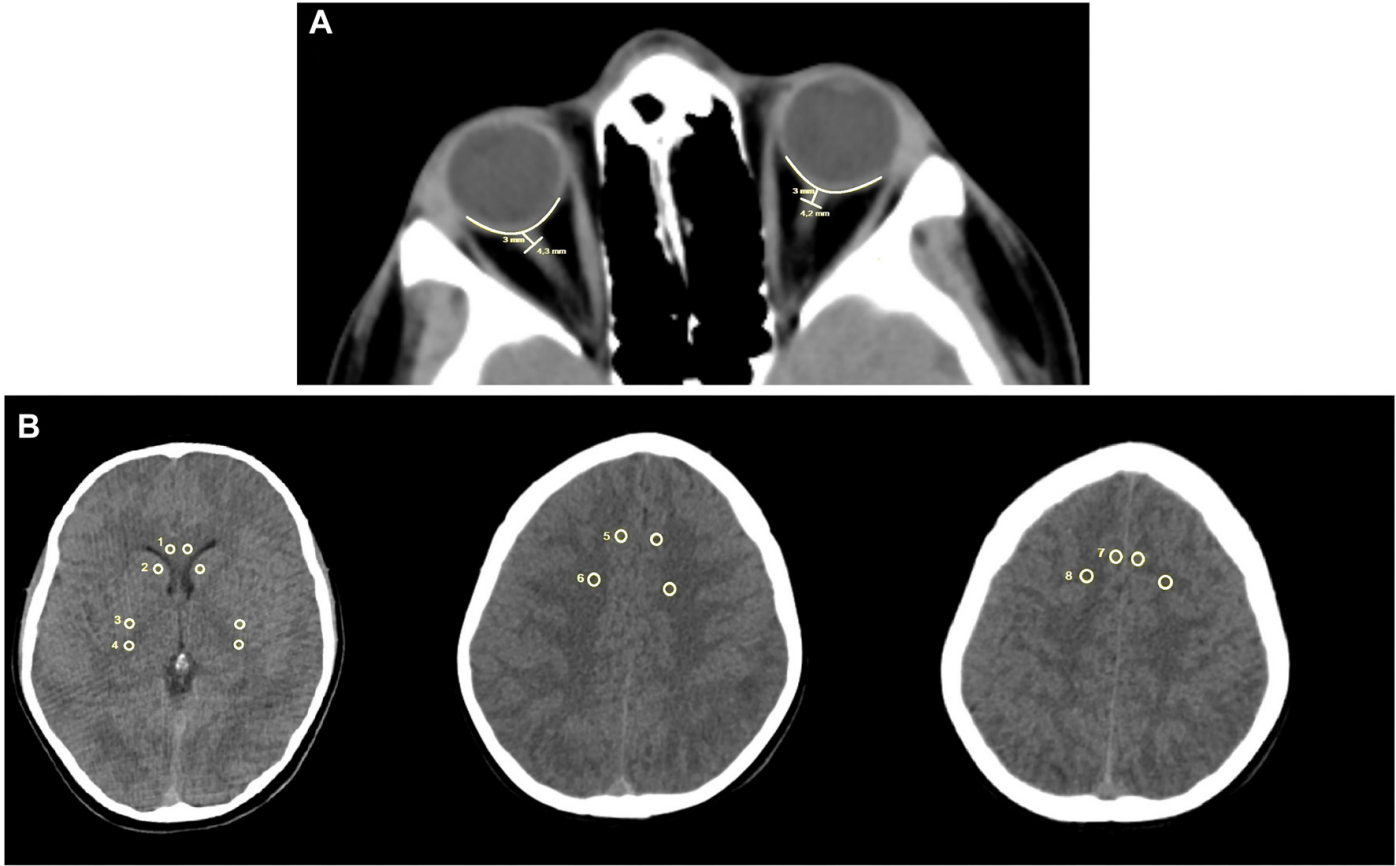
**Two samples from patients’ CT records.** (A) Measurement of ONSN with CT; (B) Measured areas at the upper cortex levels and basal ganglia level to calculate the mean GWR. GWR: Gray–white matter ratio; CT: Computed tomography; ONSD: Optic nerve sheath diameter.

ONSD values on brain CT were measured to evaluate their correlation with ultrasonographic ONSD values. In CT imaging, the ONSD was measured 3 mm behind the eyeball, immediately below the sclera, along a perpendicular vector referenced to the linear axis of the optic nerve (Figure 3) [12, 13].

Cerebral edema was assessed using the gray matter attenuation to white matter attenuation ratio (GWR) as described in previous studies [14, 15]. All measurements were evaluated by two independent radiologists who were blinded to the clinical outcomes. Only non-contrast head CT scans were included in the study. The scans were acquired using a GE LightSpeed VCT scanner with a 1-mm slice thickness (GE Healthcare, Little Chalfont, UK). Each CT scan was reviewed using imaging software (Stentor iSite, South San Francisco, CA, USA), and comparable brain slices were identified at the upper cortex and basal ganglia levels, in line with previous studies [15, 16].

Circular regions of interest (0.1–0.15 cm^2^) were placed on the designated areas, and the average attenuation in Hounsfield Units (HU) was recorded. At the basal ganglia level, bilateral attenuation values were measured for the caudate nucleus (CN), putamen (PU), corpus callosum (CC), and posterior limb of the internal capsule (PIC). The GWR for the basal ganglia was calculated using previously established methods [17] as follows:

GWR_basal ganglia_ ═ (CN+ PU)/(CC + PIC).

Similarly, bilateral attenuation values were recorded for the medial cortex (MC1) and medial white matter (MWM1) at the centrum semiovale level, as well as for the medial cortex (MC2) and medial white matter (MWM2) at the high convexity level. The GWR for the cerebrum was then calculated as follows:

GWR_cerebrum_ ═ (MC1 + MC2)/(MWM1 + MWM2)

The average GWR was calculated as follows:

GWR_average_ ═ (GWR_bazal ganglia_ + GWR_cerebrum_)/2.

Increasing cerebral edema indicates less attenuation of grey matter and a lower GWR.

### NIRS monitoring

The NIRS device measures light absorption at wavelengths of 724 and 810 nm to calculate the rSO2. For these measurements, the INVOS monitor (INVOS 5100 C; Covidien, Mansfield, MA, USA) was employed. NIRS monitoring began upon the patient’s admission to the ICU and was repeated at 24-h intervals, for a total of three measurements. Two surface sensors were positioned on the forehead to detect bilateral frontal rSO2. Data analysis was performed using the Covidien software package (INVOS Analytics Tool, Version 1.2). The mean rSO2 from the right and left sensors was calculated for analysis at each time point.

### Neurological outcome measures

Two scoring systems, one for the early stage and another for the late stage, were employed to evaluate neurological outcomes. The Full Outline of Unresponsiveness (FOUR) score was used during the early period, as reported in previous studies [18, 19]. A FOUR score greater than 6 within the first 72 h after CA was considered indicative of a good early neurological response. For late neurological outcomes, the Glasgow–Pittsburgh Cerebral Performance Categories (CPC) were utilized. CPC scores were recorded one month ROSC, with CPC 1–2 classified as a good outcome and CPC 3–5 as a poor outcome [20].

### Other data

Age, gender, occurrence of “out-of-hospital cardiac arrest” (OHCA), presence of a witness to CA event, presence of a shockable rhythm, cardiac origin of CA, time to the initiation of cardiopulmonary resuscitation (CPR), time to ROSC, time to brain CT scan, and tNIRS and ultrasonographic ONSD evaluation after CA, along with APACHE-II scores, were recorded.

### Ethical statement

This study was approved by the Health Sciences University, Hamidiye Scientific Research Ethics Committee (Decision No: 2021/1/19), was registered on clinicaltrials.gov (NCT05552794). Written informed consent is obtained from their legal representatives as all CA survivors were unconscious on admission.

**Table 1:** Baseline characteristics of study population

**Variable**		**FOUR**			**CPC**		
	***N* ═ 40**	**Good outcome *n* ═ 21 (52.5%)**	**Poor outcome *n* ═ 19 (47.5%)**	***P* value**	**Good outcome *n* ═ 14 (35%)**	**Poor outcome *n* ═ 26 (65%)**	***P* value**
Age (years)	58 (46.25–70.0)	60 (46.50--67)	56 (46--76)	0.571	57.5 (44--62.5)	58 (46.75--76)	0.241
Sex, male, *n* (%)	27 (67.5)	14 (66.7)	13 (68.4)	0.906	11 (41)	16 (59)	0.316
APACHE score	29 (26–34.75)	27 (24.50–29)	34 (30–38)	0.001*	25 (23–28.5)	32 (29–37.25)	0.001*
OHCA, *n* (%)	31 (77.5)	15 (71.4)	16 (84.2)	0.457	10 (71.4)	21 (80.8)	0.694
Shockable rhythm, *n* (%)	17 (42.5)	11 (52.4)	6 (31.6)	0.184	10 (71.4)	7 (26.9)	0.007*
Bystander CPR, *n* (%)	11 (27.5)	7 (33.3)	4 (21.1)	0.385	5 (35.7)	6 (23.1)	0.469
Collaps to NIRS/ONSD (h)	4 (3–5)	4 (2.5–5)	4 (4–6)	0.203	4 (2–5)	4 (3–5.25)	0.188
Cardiac cause of arrest, *n* (%)	18 (45)	10 (47.6)	8 (42.1)	0.726	10 (71.4)	8 (30.8)	0.014*
Time to CPR (min)	5.5 (2–10)	5 (1–10)	6 (2–12)	0.216	4 (1–8.50)	7 (2–10)	0.183
Time to ROSC (min)	16 (11–23)	16 (11–23)	25 (15–35)	0.022*	14 (10–16)	25 (15–30)	0.001*
Time to brain CT (h)	2 (1–2)	2 (1–2)	2 (1–2)	0.742	1.5 (1–2)	2 (1–2)	0.489

The data are given as median and IQR (25–75) or absolute numbers and percent. IQR: Interquartile range; OHCA: Out-of-hospital cardiac arrest; CPR: Cardiopulmonary resuscitation; ONSD: Optic nerve sheath diameter; NIRS: Near-infrared spectroscopy; ROSC: Return of spontaneous circulation; CT: Computed tomography; CPC: Cerebral performance categories.

### Statistical analysis

Statistical analyses were performed using SPSS (IBM SPSS Version 20). Data are presented as medians with interquartile ranges (IQR: 25–75) or as absolute numbers with percentages. Continuous variables are reported as medians with IQRs or means with standard deviations (SD), depending on the normality of their distribution. Categorical variables are presented as frequencies and percentages, with comparisons conducted using the chi-square test or Fisher’s exact test. Homogeneity of ONSD and NIRS values was assessed using one-way ANOVA. Point-biserial correlation analysis was performed to examine the relationship between ONSD/NIRS values and FOUR/CPC scores.

The normality of raw HU measurements and GWR values for each region of interest was tested using both visual inspection and the Shapiro–Wilk test. Additionally, binary logistic regression was used to assess associations between attenuation, GWR, and outcomes (poor vs good).

“A non-parametric longitudinal analysis was conducted to compare rSO2 values between patients with good and poor outcomes. The Spearman correlation was calculated to evaluate the relationship between NIRS values and time to ROSC. A significance level of 0.05 was applied. ROC curves were generated to assess the predictive performance of ONSD, NIRS, and the combined use of ONSD and NIRS for neurologic outcomes. Additionally, a multimodal analysis integrating ONSD and NIRS for predicting neurologic outcomes was performed using JMP Pro 13.0 (SAS Institute Inc., Cary, NC, USA).”

## Results

Of the 40 patients included in the study, 15 died during their first month of follow-up in the ICU. Within the same period, 21 patients were transferred to a palliative care unit. However, two of these patients were readmitted to the ICU within one week due to worsening conditions. The treatment of four patients continued in the ICU.

Neurological outcomes were assessed using both early (FOUR score) and late (CPC score) evaluations. Based on the FOUR score, 19 patients (47.5%) had poor outcomes (FOUR ≤6), while 21 patients (52.5%) had good outcomes (FOUR >6). According to the CPC score, 26 patients (65%) were classified as having poor outcomes (CPC 3–5), whereas 14 patients (35%) had good outcomes (CPC 1–2).

### Characteristics of study subjects

Simultaneous ultrasonographic ONSD and NIRS measurements were performed on all patients upon ICU arrival. No statistically significant difference was observed in the time from collapse to ONSD/NIRS measurements between the groups (median: 4 h [3–5]). Regarding CPC scoring, significant differences were noted between good and poor outcomes in relation to shockable rhythm and cardiac cause of arrest (*P* ═ 0.007 and *P* ═ 0.014, respectively). For both FOUR and CPC scoring systems, patients with poor outcomes had higher APACHE-II scores and longer times to ROSC (APACHE-II ═ 34; time to ROSC ═ 25 min according to FOUR; APACHE-II ═ 32; time to ROSC ═ 25 min according to CPC, *P* ═ 0.001 for all comparisons). Baseline characteristics are summarized in Table 1.

### Ultrasonographic ONSD

At the time of obtaining ONSD1,2,3 measurements, hemodynamic parameters were comparable between the groups with good and poor outcomes. A significant negative correlation was observed between ONSD1,2,3 measurements and FOUR scores (*P* ═ 0.021, *P* ═ 0.019, and *P* ═ 0.023, respectively). However, no significant correlation was found between ONSD1,2,3 and CPC scores (Table 2). In the patient who progressed to brain death, ONSD1, ONSD2, and ONSD3 values were measured as 0.78, 0.80, and 0.82 mm, respectively.

**Table 2:** Correlation of ONSD values measured at different times with FOUR and CPC

**Variable**	**Mean**	**SD**	**Point biserial correlation *r*_pb_**		***P* value**	
***N* ═ 40**			**FOUR**	**CPC**	**FOUR**	**CPC**
ONSD_1_	0.69	0.12	−0.363	0.041	0.021*	0.800
ONSD_2_	0.69	0.12	−0.369	−0.14	0.019*	0.933
ONSD_3_	0.67	0.11	−0.358	−0.046	0.023*	0.776

ONSD_1_: The first ONSD measurement performed when the patient comes to the ICU; ONSD_2_: ONSD measurement performed 24-h after the first measurement; ONSD_3_: ONSD measurement performed 48 h after the first measurement; FOUR: Full outline of unresponsiveness; ONSD: Optic nerve sheath diameter; CPC: Cerebral performance categories; SD: Standard deviations.

### Comparison of tomographic ONSD and GWR with sonographic ONSD

In order to compare the agreement between CT ONSD/GWR values and sonographic ONSD values, ultrasonographic ONSD1 measurements taken closest to the brain CT scan were used. The time interval between the collapse and the brain CT scan was not statistically different between the groups. A significant positive correlation was observed between ultrasonographic ONSD1 and CT ONSD (*P* ═ 0.018, *r* ═ 0.372). However, no significant correlation was found between ONSD1 and GWR-BG, GWR-CE, or GWR-AV (*P* ═ 0.865, *P* ═ 0.650, and *P* ═ 0.832, respectively). Additionally, tomographic ONSD demonstrated a significant correlation with both the FOUR score and CPC score (*P* ═ 0.001 and *P* ═ 0.006, respectively), whereas no correlation was found between GWR-AV and the FOURw or CPC scores (*P* ═ 0.528 and *P* ═ 0.509) (Table 3).

**Table 3:** Comparison of agreement between CT ONSD/GWR and ultrasonographic ONSD values

**Parameter**	**Mean **±** SD/Median [Range]**	***P* value**
Ultrasonographic ONSD_1_ vs CT ONSD	0.69 ± 0.12 mm (ONSD1)/0.58 ± 0.85 mm (CT ONSD)	0.018
Ultrasonographic ONSD_1_ vs GWR-BG	1.28 ± 0.12 mm	0.865
Ultrasonographic ONSD_1_ vs GWR-CE	1.31 ± 0.15 mm	0.650
Ultrasonographic ONSD_1_ vs GWR-AV	1.30 ± 0.10 mm	0.832
CT ONSD vs FOUR	−	0.001
CT ONSD vs CPC	−	0.006
GWR-AV vs FOUR	−	0.528
GWR-AV vs CPC	−	0.509

CT: Computed tomography; ONSD: Optic nerve sheath diameter; ONSD_1_: The first ONSD measurement performed when the patient comes to the ICU; GWR-BG: Gray–white matter ratio at basal ganglia; GWR-CE: Gray–white matter ratio at cerebrum; GWR-AV: Gray–white matter ratio average; SD: Standard deviation.

### NIRS (rSO2) values

**Figure 4.**
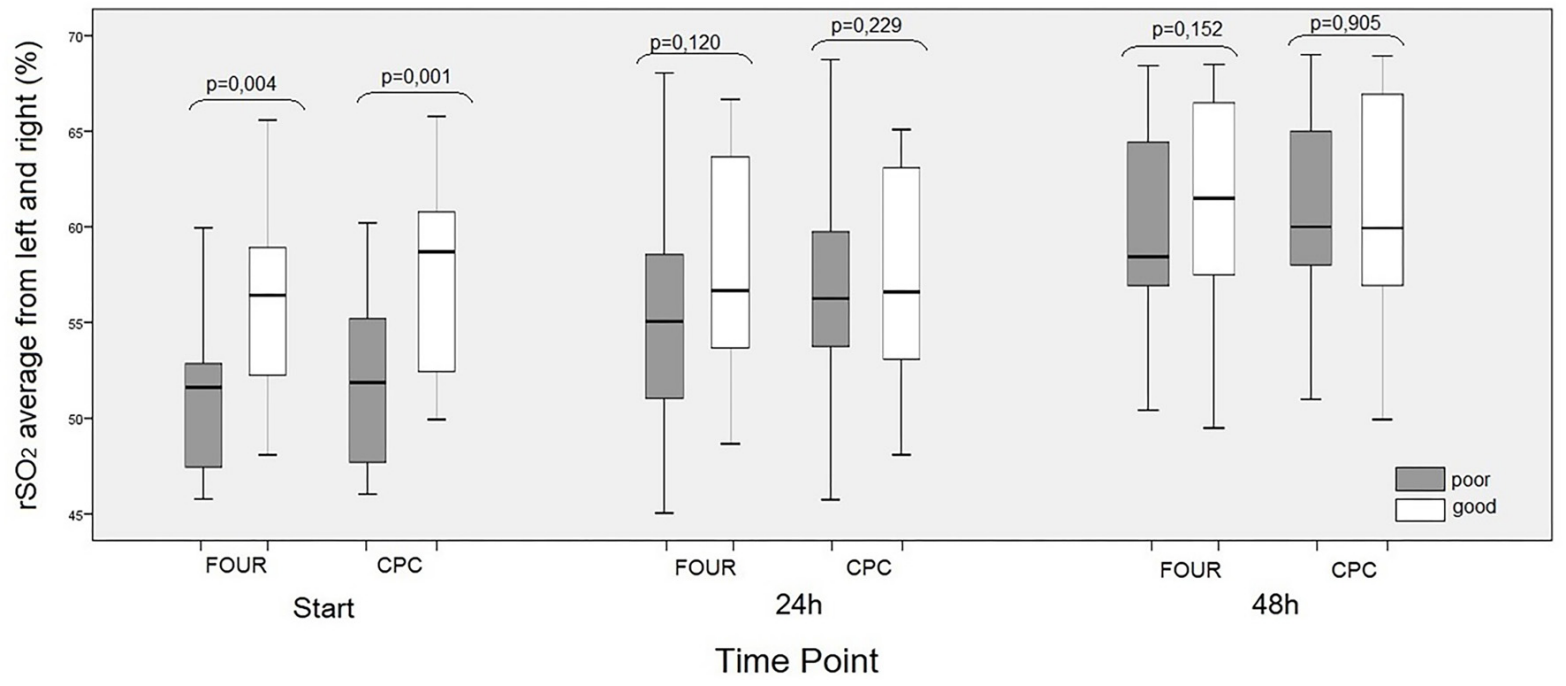
**The effects of cerebral oximeter and mean rSO2 values of right and left ONSDs on neurological outcome at baseline (NIRS_1_), 24th h (NIRS_2_) and 48th h (NIRS_3_).** Neurological outcomes were evaluated separately according to FOUR and CPC. ONSD: Optic nerve sheath diameter; NIRS: Near-infrared spectroscopy; FOUR: Full outline of unresponsiveness; CPC: Cerebral performance categories.

While there was a significant positive correlation between NIRS1 and both FOUR and CPC results (*P* ═ 0.004 and *P* ═ 0.001, respectively), no significant correlation was observed between NIRS2 or NIRS3 and the FOUR or CPC results (Figure 4).

### Outcome prediction

Since all three measures of ONSD correlated with FOUR but not with CPC, we used the arithmetic mean of these values (ONSD_AV) for statistical evaluations. According to FOUR, logistic regression analysis using ONSD_AV and NIRS1 yielded an OR of 0.001 (95% CI: 0.001–1.173; *P* ═ 0.055) for ONSD_AV and an OR of 1.198 (95% CI: 1.031–1.391; *P* ═ 0.018) for NIRS1. The sensitivity of ONSD_AV was 71.4%, with a cut-off value of 0.70 mm. For NIRS1, sensitivity was 64.3%, with a cut-off value of 52.5%. To illustrate the combined effect of ONSD_AV and NIRS1 on FOUR, we developed the following logistic regression formula:

Prob=1/[1+exp (4.756+6.5373*ONSD-0.1803*NIRS)].

**Figure 5.**
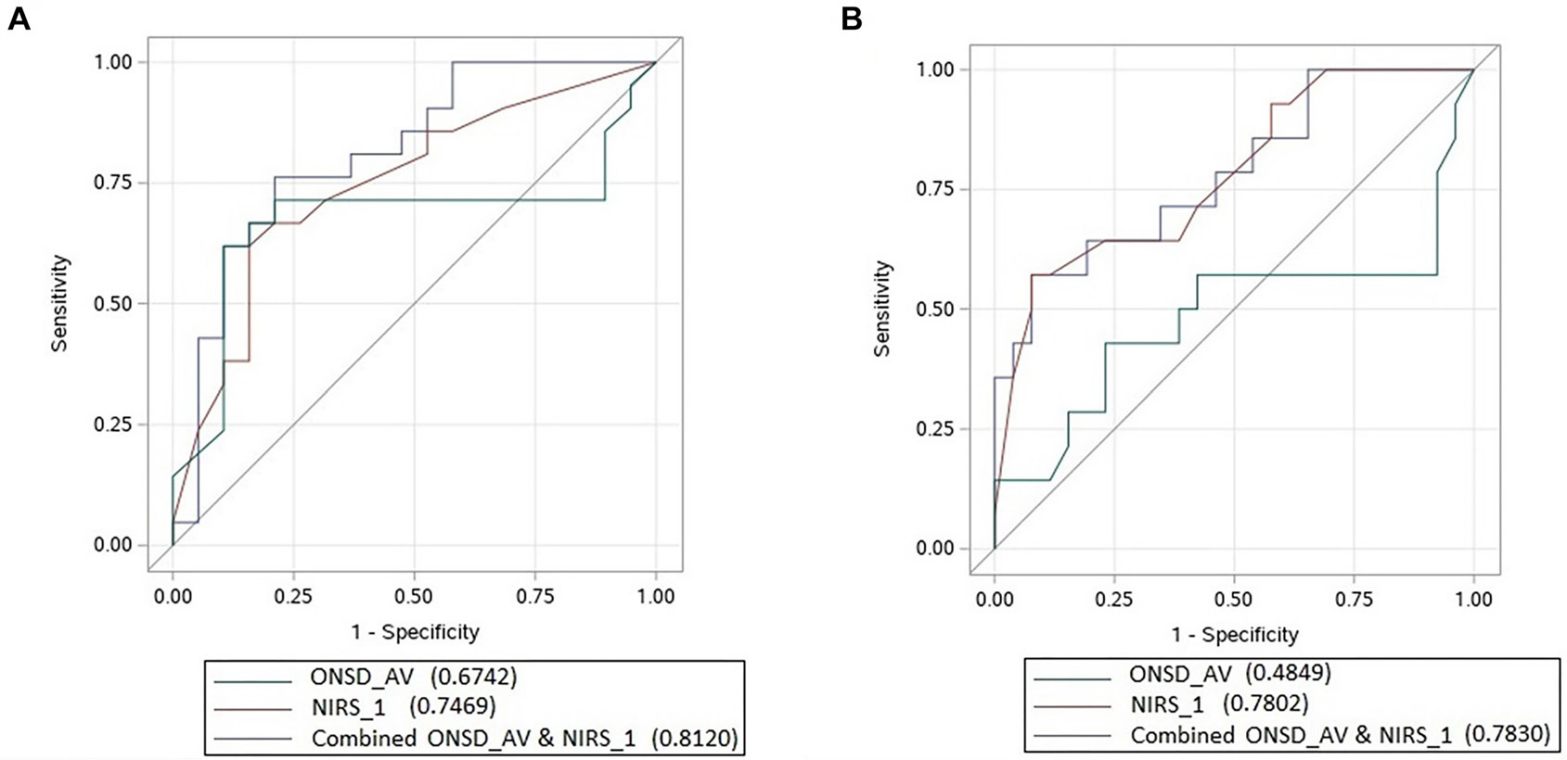
**ROC curves showing the areas under the curve of ONSD_AV, NIRS1 and combined ONSD_AV and NIRS1.** (A) The estimation of neurological outcomes evaluated according to FOUR; (B) The estimation of neurological outcomes based on the CPC assessment. NIRS_1 (NIRS_1_): The first NIRS measurement performed when the patient comes to the ICU; ONSD_AV: Arithmetic mean of all three ONSD_1,2,3_ values; ONSD: Optic nerve sheath diameter; NIRS: Near-infrared spectroscopy; FOUR: Full outline of unresponsiveness; CPC: Cerebral performance categories.

Using the formula, it can be estimated that the FOUR values will provide meaningful predictions. The AUC was 0.8120 for the combined ONSD_AV + NIRS1 model, compared to 0.6742 for ONSD_AV alone and 0.7469 for NIRS1 alone, as calculated using the logistic regression model (Figure 5A). In the evaluation based on CPC, logistic regression analysis with ONSD_AV and NIRS1 yielded the following results: for ONSD_AV, OR ═ 0.001 (95% CI: 0.001–0.731), *P* ═ 0.04; and for NIRS1, OR ═ 1.259 (95% CI: 1.073–1.478), *P* ═ 0.0048. The sensitivity of ONSD_AV was 57.1%, with a cut-off value of 0.67 mm, while the specificity of NIRS1 was 65.4%, with a cut-off value of 52.5%.

To illustrate the combined effect of ONSD_AV and NIRS1 on CPC, the following formula was developed using logistic regression:

Prob=1/[1+exp (14.3504-2.2819*ONSD-0.2306*NIRS)].

Using the formula, it can be estimated that the CPC value will be favorable. The AUC was 0.7830 for the combined ONSD_AV + NIRS1 model, compared to 0.4849 for ONSD_AV alone and 0.7802 for NIRS1 alone, as determined using this logistic regression model (Figure 5B).

## Discussion

Our study was the first to evaluate the combined effects of ultrasonographic ONSD and NIRS in predicting poor neurological outcomes in patients who achieved ROSC after CA. This novel approach adds to the existing body of research by highlighting the potential of using multiple non-invasive techniques to assess neurological outcomes in critical care settings. Specifically, the combination of ultrasonographic ONSD and NIRS may provide clinicians with a more reliable and dynamic toolset for real-time prognosis evaluation.

Our findings revealed that ONSD_AV was significantly higher, and NIRS1 was notably lower, in patients with poor neurological outcomes compared to those with favorable outcomes. Furthermore, the combined assessment of ONSD_AV and NIRS1 proved more effective in predicting poor outcomes than either method alone. These results suggest that integrating NIRS with ONSD measurement could enhance early detection of unfavorable neurological outcomes, enabling timely interventions or adjustments to patient care strategies.

In previous studies, CPC has been widely used to evaluate long-term neurological outcomes following CA [21, 22]. It remains a commonly employed method due to its clear stratification of outcomes based on levels of neurological recovery. However, a meta-analysis of 49 articles by Almojuela et al. [23] highlighted that “the existing literature favors the FOUR score as a useful outcome predictor in predicting mortality and functional outcome”. This underscores the growing recognition of the FOUR score as a valuable alternative to CPC, particularly in scenarios requiring rapid and nuanced assessments of neurological status. Many studies evaluating early neurological outcomes have utilized the FOUR score for its predictive capabilities [24–26]. Accordingly, we employed both FOUR and CPC scoring to evaluate early and long-term outcomes, aiming to provide a more comprehensive view of patient prognosis across different timeframes. This dual-scoring approach is expected to enhance the sensitivity and specificity of our predictions, offering a robust foundation for future research and clinical applications.

There are numerous studies on ONSD values and their association with neurological outcomes. The variability in findings highlights both the potential and the challenges of using ONSD as a reliable predictor. For instance, Yong et al. found that ONSD measured during the first brain CT after CA was associated with poor neurological outcomes [12], suggesting its utility as an early indicator of neurological compromise. Conversely, Lee et al. [7] reported no correlation between CT ONSD measurements and neurological outcomes. This inconsistency may stem from differences in patient populations, measurement techniques, or the timing of imaging relative to the onset of CA.

In our study, we evaluated the correlation between sonographic ONSD1 (representing the most recent CT scan) and CT ONSD. We found a positive correlation between sonographic and CT ONSD measurements. Moreover, CT ONSD was significantly associated with both the FOUR score and CPC, further supporting the value of ONSD as a prognostic tool.

Since ultrasonographic assessment of ONSD can easily be performed at the bedside in an ICU, transorbital sonography offers significant advantages over CT imaging. These advantages include the elimination of time-consuming procedures and the risks associated with patient transportation. This feature is particularly valuable in intensive care settings, where patients are often too unstable for transport. However, Park et al. [27] noted that ONSD values obtained at 24, 48, and 72 h are particularly useful for estimating neurological status. Timely and repeated measurements can highlight trends in ICP changes, offering more dynamic insights than a single static measurement. In our study, we performed ONSD measurements as soon as the patient was admitted to the ICU and repeated them at 24-h intervals twice more. This approach allowed us to monitor fluctuations potentially correlated with neurological outcomes. Chelly et al. [28] reported that the initial ONSD value at 24 h was significantly higher in non-survivors compared to survivors. Consistent with their findings, we observed that ONSD_AV values were significantly elevated in patients with poor outcomes compared to those with favorable outcomes. These results suggest a potential ONSD threshold that could help differentiate between survivors and non-survivors, underscoring the need for further research through larger, multicenter studies.

Ueda et al. [29] reported that sonographic ONSD measurement is a fast and effective technique for assessing neurological outcomes after CA. This makes it an appealing option for real-time monitoring in the ICU, where rapid assessments are critical for guiding patient care. Similarly, Chelly et al. [28] highlighted that sonographic ONSD shows promise as a tool for early evaluation of post-CA patients. In our study, we observed that higher sonographic ONSD values were associated with lower FOUR scores but not with worse CPC outcomes. This finding suggests that sonographic ONSD may be more closely linked to early indicators of neurological function, while CPC scores might reflect longer-term outcomes that are less sensitive to immediate ICP changes. The significant but weak correlation between ONSD and FOUR scores indicates that ONSD may have limited predictive value for immediate neurological outcomes, emphasizing the need for cautious interpretation.

Metter et al. [14] reported that a low GWR (<1.20) measured from early cranial CT scans after CA is associated with increased mortality. GWR is widely recognized as a marker of hypoxic brain injury, with lower values reflecting more extensive damage. Ertl et al. [30] suggested that combining sonographic ONSD and GWR could enhance prognostic accuracy. Similarly, Chae et al. [31] found that the combined assessment of CT-based ONSD and GWR improved prognostic performance, indicating these metrics may provide complementary insights. In our study, however, no significant correlation was observed between sonographic ONSD1 and GWR. Additionally, there was no significant relationship between GWR_AV and the FOUR score or CPC. This discrepancy may be attributed to technical differences between ultrasound and CT imaging methods. Ultrasonography assesses the surrounding soft tissues of the globe, while CT provides clearer anatomical boundaries. Operator-dependent factors, such as probe placement and user expertise in ultrasonography, may also have contributed to the weaker correlation. These findings suggest that although ONSD and NIRS may improve predictive accuracy, GWR might not provide additional prognostic value in this specific context.

Our previous study highlighted that high mean rSO2 values during CA are associated with favorable neurological outcomes [32]. NIRS has been increasingly adopted as a non-invasive, continuous monitoring tool in critical care, particularly for assessing cerebral oxygenation in patients at risk of hypoxic injury. Evidence from adult studies after CA suggests that rSO2 may not only aid in outcome prediction but also play a role in optimizing CPR strategies and guiding neuroprotective interventions [33]. This underscores the broader clinical relevance of NIRS beyond its utility as a predictive marker. For instance, Storm et al. reported that rSO2 levels during the first 40 h after ROSC were significantly lower in patients with poor outcomes, suggesting that early cerebral oxygenation measurements may have prognostic value. However, they also noted limited potential for predicting poor outcomes solely using frontal brain rSO2 measurements [10]. In contrast, Jakkula et al. [34] found no statistically significant difference in median (IQR) rSO2 during the first 36 h of intensive care between patients with good outcomes (70.0% [63.5%–77.0%]) and those with poor outcomes (71.8% [63.3%–74.0%], *P* ═ 0.943), although they emphasized its potential as a predictor of favorable outcomes. In our study, a significant correlation was observed between NIRS1 (the median NIRS value measured during the first 4 h after collapse) and neurological outcomes in both early and late stages of recovery. Notably, the cut-off values identified in this study—0.67 mm for ONSD and 52.5% for the first-day NIRS value—may serve as clinically relevant markers for early neurological prognosis after ROSC. However, the lack of correlation between high rSO2 values beyond the first 24 h and good neurological outcomes may be influenced by factors such as elevated FiO2 and PaO2 levels in mechanically ventilated patients. More importantly, this study specifically explored the combined prognostic utility of ONSD and NIRS measurements. Our findings suggest that while both ONSD and NIRS are valuable tools individually, their combined use provides enhanced accuracy in predicting neurological outcomes. This reinforces the potential of integrating these modalities to improve prognostic precision in post-CA care.

This study had some limitations. First, it was a single-center study with a small sample size, which may restrict the generalizability of the findings. However, the prospective design of the research is an advantage, as it allows for real-time data collection and minimizes recall bias. On the downside, the limited dataset may not produce robust predictive results.

Second, the “collapse-to-CPR” time in OHCAs may lack accuracy in some cases, as the recorded time of collapse by the patient’s relatives might differ from the actual moment of collapse. This variability could introduce inaccuracies in the data, potentially affecting the observed relationships between timing and outcomes. To address this, statistical analyses were conducted based solely on the periods documented in medical records. If the true onset of collapse could be precisely recorded, it might provide more reliable data and enhance the study’s insights into the timing of interventions as predictors of outcomes. Third, predicting poor outcomes using the (CPC) may require extended follow-up periods. Other studies have tracked patients for three to six months post-arrest, but no standard timeline has been established for assessing poor outcomes with CPC. In this study, patients were evaluated at the end of the first month, which may have been too early to capture more comprehensive neurological outcomes. A longer follow-up period, such as six months, might yield more accurate and realistic assessments. Future studies could benefit from adopting standardized and extended follow-up timelines.

## Conclusion

In conclusion, ONSD measurements and initial NIRS data from the first 24 h post-CA appear to be valuable short-term predictors of neurological outcomes in patients resuscitated after CA. Our findings suggest that while ONSD and early NIRS measurements show promising prognostic value, NIRS values beyond the first 24 h may lose predictive reliability, possibly due to external factors such as mechanical ventilation and oxygenation settings. Notably, the combination of ONSD and early NIRS measurements could enhance predictive accuracy, supporting a dual-modality approach to improve early prognosis in critical care. However, further large-scale, multicenter studies are needed to validate these findings, establish standardized measurement protocols, and refine prognostic thresholds. Future research should also explore how these tools can guide therapeutic strategies and improve patient management, ultimately enhancing both outcome prediction and treatment decisions for post-CA care.
